# Molecular miR-19a in Acute Myocardial Infarction: Novel Potential Indicators of Prognosis and Early Diagnosis

**DOI:** 10.31557/APJCP.2020.21.4.975

**Published:** 2020-04

**Authors:** Fatemeh Mansouri, Mir Hosein Seyed Mohammadzad

**Affiliations:** 1 *Department of Genetics and Immunology, Faculty of Medicine, *; 2 *Cellular and Molecular Research Center, *; 3 *Department of Cardiology, Seyedoshohada Hospital, Urmia University of Medical Sciences, Urmia, Iran. *

**Keywords:** MiR-19a, acute myocardial infarction, biomarkers, early diagnosis

## Abstract

**Objective:**

Due to the increasing annual incidence rate of disability and mortality in patients with acute myocardial infarction (AMI), the need for an appropriate diagnostic tool has become a crucial urgent issue. An increase in biomarkers and protein levels in response to AMI can be used as a predictive biomarker with different sensitivities and specificities. This study aimed at investigating the role of miR-19a as a biomarker with acceptable sensitivity and specificity for early diagnosis of AMI.

**Methods:**

We studied 175 patients with AMI admitted within 12 h of symptom onset and 90 healthy subjects as control group. Patients were divided into two groups, including group I (normal vessels and no significant artery stenosis) and primary percutaneous coronary intervention (PCI) group II (patients with more than 50% stenosis in vessels and severe atherosclerosis) diagnosed by angiography. The expression level of miR-19a was evaluated by the real-time polymerase chain reaction and other serum chemistries were also analyzed.

**Results:**

The results demonstrated that circulating miR-19a levels were significantly increased in patient groups compared to the control group (2.88 ± 1.06 vs. 5.93 ± 1.28, P<0.0001). We also found that miR-19a levels were higher in group II (134.62-fold) than group I (15.42-fold). The upper levels of miR-19a were significantly correlated with the increased serum levels of CK-MB (ρ=0.29, P<0.0001), CTn I (ρ=0.4, P<0.0001) and creatinine (ρ=0.27, P<0.0001). In addition, Receiver Operating Characteristic (ROC) analysis revealed that circulating miR-19a had considerable diagnostic accuracy for the patients with normal vessel with an AUC of 0.930 (95% CI: 0.697-0.765) and for PCI patients with an AUC of 0.966 (95% CI: 0.748-0.784).

**Conclusion:**

Circulating miR-19a possibly has prognostic value to be used as a promising molecular target for early diagnosis and prognosis of AMI.

## Introduction

Acute myocardial infarction (AMI) with increasing global annual incidence is a serious cardiovascular disease (CVD) that primarily occurs in mid to late life. It is a complex disease with several risk factors that leads to an increase in the rate of mortality and morbidity. Many risk factors, such as diet, lifestyle, smoking, air pollution, environmental and genetic factors affect the development of CVD. Due to the changes in lifestyle, decreased physical activity and diet, the global prevalence of CVD is increasing. Three main reasons for urgent hospitalization due to AMI syndrome are ST elevation myocardial infarction (STEMI), non- ST elevation myocardial infarction (NSTEMI) and unstable angina (UA) (Olivieri et al., 2014).

Today, several clinical observations, like electrocardiography (ECG), systolic blood pressure and blood laboratory parameters, such as cardiac troponin I (CTn I), creatine kinase-myocardial band (CK-MB), high-sensitivity C-reactive protein (hs-CRP) and brain natriuretic peptides (BNPs) are used for myocardial infarction (MI) (Koenig, 2013; Shrivastava et al., 2015). CTn I and other laboratory tests are considered as the acceptable routine tests in clinical settings by cardiologists for accurate diagnosis, however these test are associated with several limitations (Fichtlscherer et al., 2011). The level of CTn I can be increased 8 h after MI and it also can be raised in the liver and end-stage renal diseases, which results in a delay in diagnosis and affects the effective treatment (Li et al., 2014; Goretti and Devaux, 2016). Accordingly, these tests have no specificity and also are poor predictors. Early and timely diagnosis of AMI is crucial to improve the overall survival rate of patients in clinical practice. In addition, MI is classified in to 5 types; type 1: spontaneous MI related to a coronary plaque rupture, type 2: MI secondary to ischemia due to imbalance between oxygen demand and supply, type 3: sudden cardiac death, type 4a: MI related to percutaneous coronary intervention (PCI), type 5: MI associated with cardiac surgery (Saaby et al., 2013; Chapman et al., 2017; Thygesen et al., 2018). Percutaneous coronary intervention is an immediate procedure for treating people with AMI due to severe heart failure and muscle injury.

The elevated levels of CTn I and CK-MB can be observed in up to 30% of all PCI procedures. Since current diagnostic strategies are limited for early diagnosis of MI, noninvasive diagnostic tools and cost-effective biomarkers with specificity are urgently needed to improve MI diagnosis. A wide range of biomarkers can also be valuable for routine use in clinical settings for studying prognosis, recurrence and progression of MI and also for diagnosis patients at the high risk for MI (van Rooij et al., 2008). It has recently approved that microRNAs (miRNAs) are the major markers and ideal candidates for early diagnosis of AMI (Xiao et al., 2014). They are small non-coding RNAs of 18-24 nucleotides with the potential of regulating multiple targets and also some miRNAs can act on different targets at the same time (Mansouri, 2017). Different miRNAs inhibit gene expression by complementary sequences in the 3’-untranslated region (3’-UTR) of the target genes, of which mircroRNA-19a (miR-19a) is located on the long arm of chromosome 13 as a member of mircroRNA-17-92 cluster and its expression is associated with the development of pulmonary diseases and heart failure (Deng and Zhong, 2016). The miR-17-92 cluster consists of several miRNAs that are adjacent to each other and include miR-17, miR-18a, miR-19a, miR-19b, microRNA-20a and microRNA-92a. MiR-19a promotes tumorigenesis in some cancer types and induces apoptosis by regulating target genes. Previous studies have demonstrated that the expression of circulating miR-19a increased in patients with AMI, cardiac hypertrophy and heart failure and its circulating can be used as a unique marker. The correlation between miR-19a concentrations and MI types in emergency medical services is not clear (Liu et al., 2018). MiRNAs are stable in whole blood, plasma, urine and have the key role in cell proliferation, angiogenesis differentiation and apoptosis (De Guire et al., 2013; Mansouri, 2017). 

We hypothesized that studying the relationship between miRNAs and timely diagnosis of risk factors for AMI and expression of miR-19a has prognostic value for patients with AMI. However, the association between miR-19a expression levels in patients with MI has not been yet quantified in personalized medicine. Therefore, in the present study miR-19a levels were evaluated using real-time polymerase chain reaction (RT-qPCR) and also its sensitivity and specificity as a predictive marker was assessed by receiver operating characteristic (ROC) curve for better patient care.

## Materials and Methods

*Study design and participants*

The study population included 175 adult patients with the age range of 33-79 years who attended the Seyedoshohada Cardiovascular Medical Hospital and underwent angiography for evaluation of chest pain between December 2017 and July 2018. The level of cardiac markers, such as CTn I, CK-MB, creatinine, lipid profile were evaluated and they were subjected to commonly used laboratory tests and ECG. Only patients with the time interval of 1-12 h between symptom onset and admission were included and then coronary angiography was performed. The patients reported chest pain and had positive result of exercise tolerance testing (ETT). In subjects, ischemia and a significant stenosis were also detected by myocardial perfusion imaging (MPI) and CT-angiography, respectively. MPI can show areas of the heart muscle that aren’t getting enough blood flow and ETT is used to determine the presence of significant coronary heart disease. Early diagnosis is based on more than 50% vessels narrowing (stenosis) in at least one segment of a main coronary artery that was determined by angiography. The patients were assigned into two groups according to the results of angiography, including group I (patients with normal vessels and no significant artery stenosis) and group II (PCI) (patients with more than 50% stenosis in vessels and severe atherosclerosis). The control group included 90 subjects aged more than 35 years who had undergone a routine medical health examination and a also had not a history of MI or CVD. We tested the miR-19a level for two patients and one control groups. Also, we considered the cigarette smoking, hobble bubble consumption, addiction statues, before history for heart disease, family history for heart disease and blood group for all our subjects. In addition, patients with a history of cardiomyopathy, congenital heart disease, acute liver failure, kidney failure, hepatitis, immune deficiency disease, infections, cancer and malignant diseases were excluded from the research. We employed age and sex-matched healthy control subjects. All patients and controls agreed to participate in the study, after being informed of the goal of this research and written informed consent. The study was approved by the Medical Ethics Committee of Urmia University of Medical Sciences, Urmia, Iran (IR.UMSU. Rec. 1395. 252).

*Blood sample collection and isolation*

To evaluate miRNA expressions, 2 ml of peripheral blood was drawn by a standard procedure in K2-ethylenediaminetetraacetic acid (K2-EDTA) collection blood tubes and total miRNA extraction and cDNA synthesis was prepared using Qiagen kit (Qiagen, USA), according to manufacturer’s instructions. The purity, concentration and quality of the isolated microRNA were evaluated by Nano Drop (Nano Drop-2000C, USA). 

*Quantification of miRNA gene expression by Real-Time PCR*

Changes in miRNA genes expression were analyzed by reverse transcriptase-quantitative PCR (RT-qPCR) after reverse transcription of miRNA from each sample. First-strand cDNA synthesis (Qiagen, USA) was performed and relative quantification of miRNA was carried out using ABI StepOne Plus real-time PCR system (Applied Biosystems, USA) based on a RNA standard curve and using SYBR Green PCR master mix (Qiagen, USA). Each qPCR was performed in triplicate in 20 µl and synthetic miR-423-5p was used as the spiked-in control. PCR conditions were as follows: initial denaturation at 95°C for 10 min and 40 cycles at 95°C for 10 S and 60°C for 1 min. Gene expression analysis were carried out using comparative threshold cycle (ΔΔCT Method) as well as 2^-ΔΔCt^ methods according to the following formula: 

ΔCT _sample_ = CT _target gene_ – CT _housekeeping gene _, ΔCT _control _= CT _target gene_ – CT _housekeeping gene_, ΔΔCT=ΔCT _sample_ – ΔCT_control_ (Livak and Schmittgen, 2001). 

*Determination of CTn I and other blood chemistries*

Other blood chemistries levels in the same peripheral blood samples were measured via an AutoAnalyzer (Autolab, BT 3500, Auto analyzer Medical System, Rome, Italy) and chemiluminescence detector (CLD) (Architect i1000 SR, Abbott, USA) in the Clinical Laboratory of Seyedoshohada Cardiovascular Medical Hospital (Gholikhani-Darbroud et al., 2017). Commercial kits were used according to the manufacturer’s instructions to measure CTn I and other blood chemistries during hospitalization. The upper limit of the normal reference range was 0-1 ng/ml, 0-24 U/L and 0.6-1.3 mg/dl for CTn I, CK-MB and creatinine, respectively.

*Statistical Analysis*

Statistical analysis was performed using SPSS software version 16 (SPSS, Inc., Chicago, IL, USA), Microsoft Excel version 2010 (Microsoft Corp., Redmond, WA, USA) and XLSTAT Version 2016.02.28451 (Addinsoft, Paris, France). Values are expressed as the mean ± standard deviation (SD) for quantitative variables and comparisons between groups were analyzed with one-way ANOVA. Two-sided P values less than P<0.001 were considered as statistically significant. MiRNA levels were presented as the fold change compared to the control group. Pearson’s Correlation Coefficient was used to measure the relationships between miRNA-19a level and cardiac diagnostic tests or other blood chemistries levels. ROC analysis was conducted to construct ROC curves using the expression level of miRNA adjusted for the matching factors between different characteristics of subjects and also the area under ROC curve (AUC) was calculated. 

## Results

*Clinical characteristics of patients*

A total of 175 patients with AMI and 90 healthy subjects were enrolled in the study. The mean age of groups I and II and the control group were 55.09±10.60, 56.68± 12.25 and 53.96±8.99 years, respectively. The number of male subjects in the studied groups was greater than female subjects (male/female ratio: 38/37, 70/30 and 50/39 in groups I and II and control group, respectively). No correlation was found in geographic distribution, weight, blood pressure, sex, age and diabetes mellitus in the patients and control groups. Other related risk factors and the laboratory data between the groups are shown in Table 1.

*MiR-19a levels in the studied subjects*

**Figure 1:**
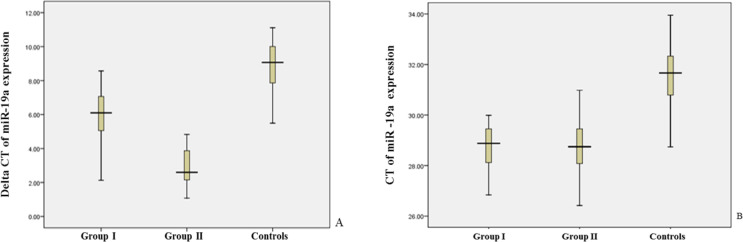
Box Plots Show miR-19a Expression Levels in AMI Patients and Healthy Subjects. Delta CT levels of the miR-19a (Fig1A) and Cycle threshold (CT) in patients and controls (Fig1B) were evaluated. Delta CT levels on the Y-axis and the groups on the X-axis are indicted. In the box plot, the thick bars show the median values of the groups. The value of the 75th percentile and the 25th percentile represent the upper lines and the lowest lines of each box, respectively

The circulating levels of miR-19a, as a marker were elevated using fold change in the AMI patients and control subjects (mean Ct=33.91± 0.81, 31.91±1.1 and 29.69±1.253 in group I, group II (PCI) and control group, P<0.001). Two patient groups showed significant differences in miR-19a levels. Its levels were 15.42-fold in group I and 134.62-fold (P<0.001) in group II. Over half of the AMI patients (73%) had Ct values of less than 30 and over half of the control group showed Ct values of more than 30. In the box plots represent *miR-19a* expression levels in patients and control subjects and the relative expression values are presented on the Y-axis (Figure 1A, B).

*The correlation between miR-19a levels and other blood chemistries*

The correlation between expression levels of miR-19a and blood chemistries were analyzed. No significant differences were detected between miR-19a levels and gender, age, ethnicity, weight, systolic/diastolic blood pressure, Fasting blood sugar (FBS), serum Na and K, white blood cells (WBC), red cell distribution width (RDW), mean platelet volume (MPV), mean cell volume (MCV), mean corpuscular hemoglobin (MCH), mean corpuscular hemoglobin concentration (MCHC) and platelet levels in the studied population. We also performed Pearson’s correlation to evaluate the correlation between miR-19a levels and other examinations. Furthermore, circulating levels of miR-19a were positively correlated with the concentrations of CTn I, CK-MB, creatinine, triglyceride, serum Mg, low-density lipoprotein (LDL), high-density lipoprotein (HDL) (negative correlation) and the levels were weakly correlated with the concentrations of urea and cholesterol in AMI patients. The plasma levels of CTnI and the correlation between miR-19a and plasma levels of CTn I, CK-MB, triglyceride and creatinine were significantly higher in the primary PCI group than the patients group I (Table2).

*MiR-19a expression as a potential predictor of acute myocardial infarction: ROC analysis*

**Figure 2:**
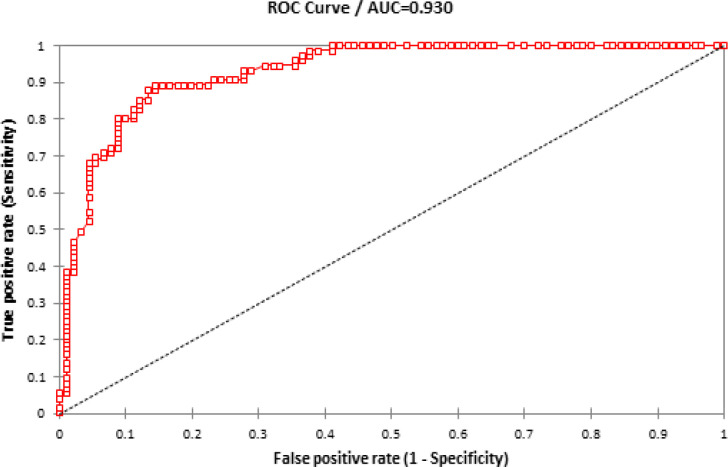
The ROC Analysis was Conducted to Investigate the Performance of miR-19a Expression Levels in Group I Patients

**Figure 3:**
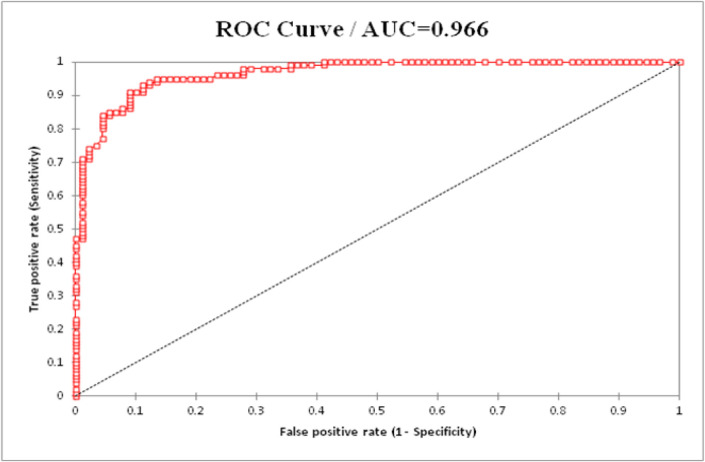
The ROC Analysis was Conducted to Investigate the Performance of *miR-19a* Expression Levels in Group II (PCI) Patients

**Figure 4:**
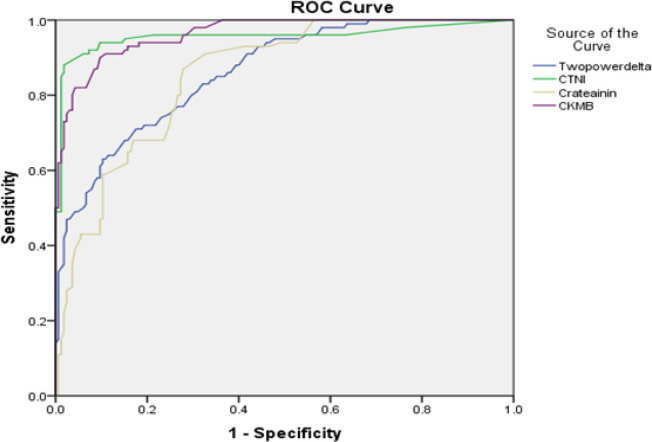
The ROC Analysis was Conducted to Investigate the Performance of miR-19a Expression Levels with UC (0.86), CTNI (O.95), Crateainin (0.85) and CK-MB (0.96) in group II (PCI) Patients

To evaluate the sensitivity and specificity of circulating miR-19a as a predictive biomarker for AMI, ROC analysis was performed for AMI patient groups. Comparing group I and control group showed that the AUC was 0.930 (95% CI = 0.697–0.765, P < 0.0001) with high sensitivity (0.91), specificity (0.856) and accuracy (0.883) (Figure 2). In addition, comparing group II (PCI) and control group indicated that AUC was 0.966 (95% CI = 0.748–0.784, P < 0.0001) with high sensitivity (0.95), specificity (0.87) and accuracy (0.91) (Figure 3). Moreover, a comparison was made to investigate the performance of *miR-19a* expression levels with AUC (0.86), CTnI (0.95), creatinine (0.85) and CK-MB (0.96) in primary PCI group (Figure 4).

**Table 1:** Comparison of Quantitative and Qualitative Variables between Groups

Variables	Group I patients	Group II (PCI) patients	Healthy controls Mean ±SD	*P*-value
	Mean ±SD	Mean ±SD		
Age (years)	55.09± 10.60	56.68± 12.25	53.96±8.99	0.001
Gender, male	38(50.7%)	70(70%)	51(56.7%)	-
Weight (kg)	80.59±13.01	84.09± 13.16	78.42±9.38	0.001
Systolic Pressure (mmHg)	124.08±1.8	136.59± 2.3	124.11±1.7	0.001
Diastolic Pressure (mmHg)	78.56±0.9	82.00± 1.1	79.83±0.9	0.001
History of heart disease (Positive)	23(30%)	38 (38%)	16(17.8%)	-
Family history of heart disease (Positive)	6.36±2.26	6.70±2.05	6.67±1.59	-
Cigarette smoking (Positive)	33(44%)	53(53%)	31(34.4%)	-
Hubble bubble(Positive)	17(22.7%)	23(23%)	7(7.8%)	-
FBS (mg/dl)	98.16±22.01	100.95± 24.62	89.18±10.47	0.59
CTN I(ng/ml)	0.62± 0.7	4.14±2.29	0.08±0.41	0.001
Urea (mg/dl)	33.28± 12.5	36.04± 13.78	29.47±7.62	0.05
Creatinine (mg/dl)	1.22± 0.60	1.56± 0.53	0.99±0.15	0.001
Creatinine Kinase-MB (U/L)	32.97± 15.93	164.06± 139.31	16.93±3.44	0.001
Blood Sugar (mg/dl)	109.43±40.57	147.30± 55.06	98.98±7.7	0.001
Serum Na (mEq/L)	140.43± 3.28	137.54±18	140.93± 3.19	0. 01
Serum K (mEq/L)	4.07± 0.4	4.22± 0.44	3.99 ±0.42	0. 01
Serum Mg(mg/dl)	2.34± 0.28	2.44± 0.38	2.03±0.19	0. 01
Cholesterol (mg/dl)	147.07± 37.76	163.84± 46.42	128.16±31.64	0.025
Triglyceride (mg/dl)	122.09± 60.12	163.84± 46.42	121.02±31.47	0.001
HDL (mg/dl)	50.68±12	43.82± 12.88	70.69±11.38	0.001
LDL (mg/dl)	99.11± 33.05	110.35± 41.96	70.54±14.21	0.001
SGPT (ALT) (mg/dl)	33.28±21.87	78.42± 115.57	26.55±7.73	0.001
SGOT(AST) (mg/dl)	30.61± 6.83	79.57±115.54	26.56±7.71	0.001
W.B.C (×1000/mm^3^)	8.04± 2.08	9. 87± 2.94	8.33±1.43	0.001
RDW (%)	13.51± 1.21	13.63± 1.15	13.17±1.89	0.005
MPV(fL)	9.49± 1.19	9.22± 0.88	9.53±1.41	0.006
M.C.V(fL)	90.12± 3.97	89.97± 4.49	89.73±3.53	0.001
M.C.H (Pgm)	29.50± 2.16	30.05± 2.60	29.36±1.83	0.84
M.C.H.C (%)	36.77± 35.04	33.36± 1.33	32.61±1.51	0.001
Platelet (×1000/mm^3^)	244.51± 88.77	247.12± 83.4	275.62±84.84	0.001
HbA1C (%)	4.84±1.32	5.27± 1.72	Not determined	0.05
MiR-19a CT	33.91± 0.81	31.91± 1.101	29.69±1.253	0.34
miR-423-5p CT	27.98± 0.77	28.97± 1.16	20.78±1.25	0.62
Mean of Delta CT	1.189± 0.302	0.466± 0.337	2.563± 1.147	-
ΔΔCT	-2.88±1.06	-5.93±1.28	8.91±1.41	-
Fold change 2^ -ΔΔCT^	15.42±21.8	134.62±16.92	-	-

Data are showed as the mean with the standard deviation (SD) and count (%). Delta CT, Difference between mean CTs of experimental and housekeeping gene for patients, cTnI, Cardiac troponin I; CK-MB, creatine kinase-myocardial band; Na+, sodium; K+, potassium; TG, Triglyceride, LDL, low-density lipoprotein; HDL, High density lipoprotein; AST, aspartate transaminase; ALT, Alanine transaminase; W.B.C, white blood cells; FBS, Fasting blood sugar; MCV, Mean corpuscular volume; MCH, Mean corpuscular hemoglobin; MCHC, Mean corpuscular hemoglobin concentration; RDW, Red cell distribution width.

**Table 2:** Correlation Analysis (Pearson’s) between Some Variables and miR-19a Fold Change that was Statistically Different among groupII (PCI) Patient, Group I Patient and Controls

Variables	Pearson	R^2^	*P*-values	Pearson	R^2^	*P*-values
	GroupII (PCI ) Patients			GroupI patients		
FBS	**0.19**	0.04	**0.005**	0.134	0.01	0.08
CTNI	**0.4**	0.16	**<0.0001**	**0.214**	0.04	**0.006**
Urea	**0.36**	0.02	**0.04**	0.49	0.002	0.53
Creatininee	**0.36**	0.13	**<0.0001**	0.061	0.004	0.43
CK-MB	**0.25**	0.06	**0.000**	0.103	0.01	0.18
BS	0.23	0.05	0.001	0.03	0.001	0.65
Serum Na	-0.054	0.003	0.44	0.09	0.009	0.21
Serum K	0.1	0.01	0.15	0.08	0.007	0.27
Serum Mg	**0.29**	0.09	**<0.0001**	**0.19**	0.031	**0.01**
Cholesterol	**0.17**	0.03	**0.01**	**0.17**	0.03	**0.02**
Triglyceride	**0.3**	0.09	**<0.0001**	**0.206**	0.042	**0.008**
HDL	**-0.43**	0.18	**<0.0001**	**-0.21**	0.047	**0.005**
LDL	0.2	0.04	**0.003**	-0.02	0.001	0.72
ALT	0.01	0	0.84	0.02	0.001	0.76
AST	0.2	0.07	0.0001	0.07	0.005	0.36
W.B.C	0.05	0.003	0.47	-0.05	0.003	0.47

Bold numbers represent significant correlation; Pearson’s r, Pearson’s Regression; R^2^, Coefficients of Determination

## Discussion

AMI is a common leading cause of death and also one of the most reasons for death-related mortality worldwide. It is crucial to explore new diagnostic biomarkers and prognostic options. A large number of studies have shown the role of miRNAs in some diseases (Biasucci and Cardillo, 2013). In these studies, miRNAs have shown to be aberrantly expressed in the different processes, such as differentiation, motility, angiogenesis; tumorigenesis, metastases and inflammation and changes in miRNAs expression levels can be detected in patients and healthy individuals. Several biomarkers in cerebrospinal fluid (CSF), plasma, blood or urine samples can be used for targeted therapies. Several studies have been done on the possible molecular mechanism of MI (Zhang et al., 2013). The role of molecular markers as prognostic indicators in MI and also the relationship between miRNAs (as convenient treatment options and being minimally invasive procedures) and risk factors for AMI have widely considered by researchers (Matsumoto et al., 2012). There are strong correlations between the clinical and molecular assays in early diagnosis and less-invasive and reliable diagnostic procedures, depending on the situation. Recent studies reported that the most focused cardiac-specific miRNAs are microRNA-208b-3p, microRNA-499a-5p, microRNA-133 and microRNA-1 (Olivieri et al., 2013; Wang et al., 2013; Olivieri et al., 2014; Yao et al., 2014; Cortez-Dias et al., 2016). It has been found that microRNA-133a-3p levels are associated with myocardial salvage, infarct size and microvascular obstruction (Deng and Zhong, 2016). MiR-19a is stable in the blood, plasma, urine and plays a key role in cell proliferation, angiogenesis, differentiation and apoptosis (Wang et al., 2018). 

Our results showed that miR-19a levels rapidly increased at the onset of cardiac symptoms compared to the control group, whereas in PCI patients, miR-19a levels were increased in the cardiac-specific miRNAs levels compared to the group I. MiR-19a levels were approximately 134.62-fold higher in primary PCI group II and increased the susceptibility to AMI. This result is clinically valuable, because these small biomarkers were released into the blood circulation as a result of myocardial damage and had a high potential to improve clinical diagnostic decision-making (Lippi et al., 2013). Our findings are consistent with the results of previous studies. Zhong et al., (2014) showed that circulating miR-19a levels were higher in the AMI patients compared to the control group in a Chinese population. In the current study, the appearance of miR-19a in circulation in AMI patients suggested that the release of miR-19a is positively associated with the extent of coronary stenosis and also the expression levels of miR-19a in patients with three-vessel coronary artery narrowing were significantly than those with stenosis of one and two vessels (less than 50% stenosis). These results suggest that *miR-19a* is expressed in cardiac muscle cells in response to stress due to inflammation, stenosis or apoptosis. The increased miR-19a is highly restricted to the degree of coronary atherosclerosis, heart failure or it may affect the progression of atherosclerosis in blood circulation of patients, which can be explained by circulating miR-19a that is extremely stable (Deng and Zhong, 2016). All samples were enrolled in less than 12 h of the onset of symptoms. 

In patients with MI, cTn I and CK-MB are the gold standards for diagnosis of MI in the heart clinics. The concentration of plasma cTn I was significantly different among two patient groups. The results showed that the levels of miR-19a were positively correlated with the severity of coronary artery stenosis in MI patients. These findings indicated that the elevated miR-19a levels in blood compared to cTn I more appropriately demonstrated the severity of MI in patients. These findings are consistent with previous studies. Ward et al. have revealed that several miRNAs were differentially expressed across plasma, platelets and leukocytes in patients with NSTEMI and STEMI and its regulation is important for the pathogenesis of acute coronary syndrome (ACS) (Ward et al., 2013). The expression of miR-19a is highly associated with cardiomyocytes and it has been shown to be present in the blood circulation of patients with AMI. 

ROC analysis was conducted to investigate the performance of miR-19a. MiR-19a can be considered as a more effective biomarker (AUC=0.966) in primary PCI patients than those with normal vessels (AUC=0.930). MiR-19a exhibited a higher sensitivity (95%) in primary PCI patients group than those with normal vessels (91%). In addition, the specificity of miR-19a in primary PCI patients (87%) was higher than that of patients with normal vessels (85%). It was estimated that some patients (about 64%) do not have chest pain or other symptoms. It was possibly predicted by monitoring miRNAs in the samples collected at the time of the initial heart attacks. In addition, the use of miR-19a in different types of MI may act more effectively than traditional tests for patients with few symptoms at early stages of MI for strict diagnostic approaches. Therefore, predictive biomarkers for the development of targeted therapies and novel therapeutic methods for early diagnosis of AMI should be identified (Goretti and Devaux, 2016).

The elevated expression of miR-19a compared to the low expression level in control subjects, which can be associated with the heart attack has the potential to provide the useful prognostic value to predict the obtained tests results in clinical care.

In conclusion, based on the results, the more appropriate MI management and also successful treatment is achievable. The increased expression of miR-19a was observed in patients in our study indicated the susceptibility to MI. It is important for both therapeutic and diagnostic strategies in the future which require further examinations for replacing troponin, CK-MB and other traditional tests for personalized medicine to the standard treatment protocols and enhance therapeutic success. Accordingly, anti-miRNA-19 can be a new target in treatment of MI with regulating the effect of some drugs, and will provide a new procedure for treatment of MI.

## Data Availability

All data during the current study are available from the corresponding author on reasonable request.
